# Mapping How Objective Structured Clinical Examinations Are Operationalized to Support Student Learning Across Nursing, Nurse Practitioner, and Medical Education: Protocol for a Scoping Review

**DOI:** 10.2196/93899

**Published:** 2026-08-12

**Authors:** Lisa Creelman, James Lomen, Cheltey Berlinguette, Natalie Boersma, Amy Beck

**Affiliations:** 1 School of Nursing, Thompson Rivers University, 840 College Drive, Kamloops, BC, V2C 0C8, Canada, 1 250-371-5532; 2 Faculty of Health Disciplines, Athabasca University, Athabasca, AB, Canada

**Keywords:** education, medicine, nurse practitioner, nursing, objective structured clinical examination, OSCE, simulation

## Abstract

**Background:**

Objective structured clinical examinations (OSCEs) are a common assessment tool used to evaluate the application of clinical knowledge, clinical reasoning, competency-based performance, and professional behavior in health care education. Their development and facilitation are often included under the broader umbrella of simulation pedagogies. Despite their widespread adoption and the availability of guidelines, variability exists in approaches specific to OSCE development, implementation, and evaluation.

**Objective:**

The purpose of this scoping review is to explore and describe the variability and similarities in OSCE development, implementation, and evaluation in nursing, midwifery, and medical education in the context of existing guidelines to inform future directive work in this area.

**Methods:**

This review will follow the JBI methodology for scoping reviews, with searches conducted by a librarian familiar with the scoping review process. Records retrieved will be reported in accordance with the PRISMA-ScR (Preferred Reporting Items for Systematic Reviews and Meta-Analyses extension for Scoping Reviews). Two reviewers will independently assess the records for inclusion against the defined criteria and extract data using an adapted JBI extraction form. The data will be charted using narrative summary text, tables, and figures.

**Results:**

As of July 2026, a search for existing reviews in the PubMed, PROSPERO, Cochrane Database of Systematic Reviews, and JBI Evidence Synthesis databases has been conducted, yielding no current or in-progress reviews on our specific topic. An initial search feasibility assessment has been conducted to ensure sufficient articles for a robust review. Data collection, extraction, and analysis will be conducted over the 12 months following the literature search. The completed scoping review is anticipated to be submitted for publication in summer 2027.

**Conclusions:**

This review will provide a comprehensive overview of how OSCEs are operationalized across nursing, midwifery, and medical programs. It will highlight emerging themes, key differences between guidelines and practice, and underresearched areas, informing future research and educational practice.

## Introduction

Health care education provides students with the foundational knowledge and skills needed to perform “accurate diagnosis, effective treatment planning, and the delivery of high-quality patient care” [1]. Objective structured clinical examinations (OSCEs) are defined as “[a]n assessment tool based on the principles of objectivity and standardisation, in which the candidates move through a series of time-limited stations in a circuit for the purposes of assessment of professional performance in a simulated environment. At each station candidates are assessed and marked against standardised scoring rubrics by trained assessors.” [2]. OSCEs are widely recognized as an essential tool for assessing the application and demonstration of knowledge, clinical competence, and professional behavior in health care education by evaluating student performance on complex tasks in standardized simulated practice scenarios [3].

Following their first introduction in the education literature, OSCEs have had a progressive evolution of guidance available for implementation. Harden et al [4] were the first to describe an evaluative process of using rotating clinical stations, later defined as OSCEs. Guiding principles for best practice in OSCE delivery were compiled and presented by Nulty et al [5] in 2011, offering OSCE developers 7 best practice guidelines providing an underlying structure upon which to build these examinations. Simulation best practice guidelines evolved along a similar timeline, with the International Nursing Association for Clinical Simulation and Learning (INACSL) publishing its first best practice guidelines related to simulation in 2011 [6]. The cornerstone of these recommendations contains guidance related to professional integrity, prebriefing, facilitation, and debriefing [7]. As OSCE delivery falls within the pedagogy of simulation, these standards have relevance in OSCE design. These two sets of guidelines became interrelated, as Nulty’s guidelines [5] were subsequently validated by multiple authors [8,9], with later authors citing INACSL guidelines as evidence of the validity of Nulty’s work [8]. Current education guidance strongly links INACSL best practices as core elements of OSCE development and use [3]. Additionally, OSCEs regularly use simulated patient actors to ensure realistic, consistent, and replicable performances across multiple simulations, thereby facilitating objective and equitable evaluation approaches [3]. The Association of Standardized Patient Educators (ASPE) has developed best practice standards with clear guidance for creating safe and objective simulation experiences that achieve the intended learning outcomes [10].

While these foundational elements are readily available to educators, concrete guidance on the effective application of these interrelated standards in the development and implementation of OSCEs appears to be elusive in the literature. There is ample description of significant variability in how OSCEs are operationalized across health care education programs [11,12], which may compromise the wider validity and reliability of these competency-based assessments. Although variation in OSCE operationalization is inevitable and may be required to meet varying operational or educational needs, the key foundation that differentiates OSCE simulations from other simulations is that they are rooted in the objectivity and consistency of their design, supported by standardized scoring tools [3]. The need to address this gap has intensified with recent national regulatory changes to nurse practitioner (NP) practice in Canada, which have shifted to generalist entry-to-practice competencies [13]. This shift requires educational programs to demonstrate how their assessment methods effectively evaluate broad competency indicators for health care provision across all ages and practice settings.

The purpose of this review is to explore and describe the variability and similarities in OSCE development, implementation, and evaluation in undergraduate and graduate nursing, midwifery, and medical education in the context of existing guidelines to inform future directive work in this area. The specific question this scoping review intends to answer is as follows: How are OSCEs used and evaluated in nursing education, midwifery, and medical education to meet course and program competency-based learning outcomes? Our subquestions include the following: (1) How are OSCEs designed and structured across these programs? (2) What evaluation methods are used to assess OSCE effectiveness? (3) What challenges and facilitators are identified in OSCE implementation?

## Methods

The proposed scoping review will be conducted in accordance with the JBI methodology for scoping reviews [14] and reported in line with the PRISMA-ScR (Preferred Reporting Items for Systematic Reviews and Meta-Analyses extension for Scoping Reviews) [15]. This protocol has been registered on the Open Science Framework [16].

### Search Strategy

#### Search for Existing Reviews

A search of the PROSPERO, PubMed, Cochrane Database of Systematic Reviews, and JBI Evidence Synthesis databases was conducted to review existing scoping or systematic reviews on the topic to avoid duplication. No current or in-progress reviews on our specific topic were identified. The authors identified 3 noteworthy systematic reviews that examined OSCE simulations: the first focused on virtual OSCEs (vOSCEs), the second on the effectiveness of OSCEs in increasing NP knowledge and skill competencies, and the third explored the use of OSCE in undergraduate nursing programs and students’ perceptions of OSCE effectiveness.

The first systematic review, conducted by Chan et al [17], examined the experiences of students and faculty in vOSCEs within the context of health professions education. vOSCEs are defined within this review as OSCEs with modifications that allow for testing to occur through online videoconference platforms rather than traditional in-person OSCEs [17]. The findings demonstrated complex technical and nontechnical challenges and barriers to consider when designing and implementing vOSCEs, while also highlighting positive unintended outcomes, such as strengthening student confidence in providing care in a virtual environment [17]. The authors posit that vOSCEs have validity as a supplemental modality for competency-based assessment as they bridge the disconnect between traditional educational assessment strategies and the realities of clinical practice [17]. The review briefly discusses examiner and station calibration sessions, as well as the recruitment of standardized patients to promote consistency of patient behavior [17]. This systematic review provides some insight into the development, implementation, and evaluation of vOSCEs; however, these findings cannot be extrapolated to other methods of OSCE assessment.

The second systematic review, conducted by Sibley et al [18] examined the effectiveness of OSCEs and simulated patient simulations in enhancing learner knowledge (clinical competence) and skill acquisition compared with alternative teaching and learning methods in family NP education. The results of this review yielded no studies meeting the inclusion criteria, underscoring the lack of high-quality evidence on how OSCEs are operationalized to enhance NP knowledge and skill acquisition. These findings provide justification for evaluating how health care education programs operationalize OSCEs to achieve competency-based learning outcomes. The objectives of our proposed scoping review are broader in scope, seeking to assess how OSCEs are developed, facilitated, and evaluated in health care education, expanding the scope and population used in the Sibley et al [18] review.

In the third systematic review, Kassabry [19] examined 18 articles exploring the use and effectiveness of OSCEs within undergraduate nursing programs, reporting on student perceptions of OSCEs completed on high-fidelity mannequins compared with traditional evaluation methods. Kassabry [19] reported OSCEs to be widely used in nursing education, perceived as fair and reliable in evaluating undergraduate nursing student knowledge and skills, and associated with increased self-confidence, independence, and improved practical and clinical skills. The work by Kassabry [19] provides another foundation for our review, upon which we aim to explore further scholarly contributions and implications beyond undergraduate nursing students using high-fidelity simulation while simultaneously adding to this work by capturing further literature published in the 3 years beyond this publication.

#### Assessment of Search Feasibility

An initial, limited search of the MEDLINE (EBSCOhost) database was conducted to identify articles on the topic and ensure that the search yielded a sufficient number of records for a scoping review. The authors consulted a librarian familiar with scoping reviews to develop a full search strategy. The text words contained in the titles and abstracts of relevant articles, and the index terms used to describe the articles, were used to develop a full search strategy in MEDLINE (EBSCOhost; Table 1). The authors randomly reviewed 50 articles to screen titles and abstracts to ensure the search strategy would provide enough articles for this review.

**Table 1.** Preliminary search strategy for the MEDLINE Complete (EBSCOhost) database.

Search^a^	Query	Records retrieved
1	(MH “Education, Nursing+“) or (MH “schools, medical”) or (MH “schools, nursing”) or (MH “faculty, medical”) or (MH “faculty, nursing”) or (MH “students, medical”) or (MH “students, nursing”)	194,508
2	((MH nurses+) or (MH nursing+) or (MH physicians+) or (MH medicine+) or (MH “physician assistants+”)) and ((MH education+) or (MH faculty+) or (MH “ED”))	290,588
3	XB((doctor or doctors or physician* or feldsher* or medical or medicine or nurs* or NP or midwi* or BSN or RN or APRN or BSCN or MSN or MSCN or RPN) N2 (student* or pupil or train* or learn* or teach* or examinee* or educat* or degree* or program* or bachelor* or baccal* or school* or universit* or undergrad* or college* or facult* or instruct* or curricul* or syllab* or pedagog*))	496,930
4	S1 OR S2 OR S3	747,842
5	XB((“objective structured” or “objective simulat*”) N2 (exam* or assessment* or encounter*))	4265
6	XB(OSCE or OSCEs or OSPE^b^ or OSPEs or VOSCE or VOSCEs or OSATS^c^ or OSVE^d^ or OSVEs or TOSCE^e^ or TOSCEs or “student performance appraisal*”)	4293
7	S5 OR S6	5480
8	S4 AND S7	3916
9	PT “Editorial” OR PT “Comment” OR PT “News” OR PT “Newspaper Article”	1,811,768
10	S8 NOT S9	3872

a Search conducted on November 30, 2025; no search limits applied.

b OSPE: objective structured practical examination.

c OSATS: objective structured assessment of technical skills.

d OSVE: objective structured virtual examination.

e TOSCE: team objective structured clinical examination.

#### Protocol Search Strategy

This scoping review will consider quantitative, qualitative, and mixed methods study designs for inclusion. In addition to published primary studies, this review will consider systematic reviews to locate primary studies, dissertations, and gray literature (prepublished, non–peer-reviewed articles). When both peer-reviewed and gray literature versions are available, peer-reviewed publications will be prioritized for data extraction. Conference abstracts, editorials, opinion papers, and protocols will be excluded because of the inability to extract study parameters and outcome data. During title and abstract screening, non-English titles and abstracts with translations that meet the inclusion criteria will be included for full-text review. Titles and abstracts without translation will be removed as reviewers will not be able to determine if they meet the inclusion criteria. Full-text non–English-language publications without translation will be collected but excluded from the review due to financial resource implications inherent in translation. This review will record the number of articles and languages so that readers can identify and retrieve the articles for further review.

The search strategy, including all identified keywords and index terms, was developed in consultation with a health sciences librarian with experience in systematic searches during the feasibility search. The reference lists of included articles will be screened for additional papers. Articles will include publications from database inception to provide depth to the review. The databases to be searched include CINAHL (EBSCOhost), ERIC (EBSCOhost), MEDLINE (EBSCOhost), Education Research Complete (EBSCOhost), and Scopus. Sources of unpublished studies and gray literature to be searched include Google, Sigma Repository, the Agency for Healthcare Research and Quality, and EdArXiv.

### Inclusion and Exclusion Criteria

This review will include studies with participants enrolled in prelicensure undergraduate and graduate nursing (registered nurse, psychiatric nurse, and NP students); midwifery; and medical education (medical and physician assistant students) programs. As some Canadian provinces provide separate programs and licensure recognition for registered psychiatric nurses [20], psychiatric nursing students have been included in the population criteria. Studies that report on OSCEs used within postgraduate and postlicensure contexts and workplace practice settings will be excluded. Furthermore, studies that report on other health-related professions (ie, health care aides, social work, physiotherapy, and respiratory therapy) will also be excluded. No limitations will be applied to the geographic location of published studies.

Studies that describe the educational content and characteristics or standards of the design, development, implementation, or evaluation of OSCEs will be included. We will consider OSCEs of any length or duration. This review will exclude studies that use OSCEs solely as a metric to evaluate specific pedagogies or different learning modalities, as this does not further knowledge of the operationalization of the OSCE itself.

### Study Selection

Following the search, all identified records will be collated and uploaded into Covidence, an online platform for managing systematic reviews, and duplicates will be removed before screening. Titles and abstracts will then be screened by 2 independent reviewers to assess compliance with the review’s inclusion criteria. Reviewers will screen 100 articles and meet to review any conflicts to calibrate their understanding of the application of the inclusion and exclusion criteria. Any disagreements that arise between the reviewers at each stage of the selection process will be resolved through discussion or consultation with a third reviewer. Potentially relevant papers will be retrieved in full, and their citation details will be imported into Covidence. The full text of selected citations will be assessed in detail against the inclusion criteria by 2 independent reviewers. Reasons for exclusion of full-text papers that do not meet the inclusion criteria will be recorded and reported in the scoping review. The results of the search will be reported in full in the final scoping review and presented in a PRISMA (Preferred Reporting Items for Systematic Reviews and Meta-Analyses) flow diagram [21].

### Data Extraction

Data will be extracted from included papers by 2 independent reviewers using a data extraction tool developed by the reviewers following JBI best practice standards to capture key concepts related to the scoping review’s focus. The data extracted will include specific details about the authors, publication year, source, study type, description of OSCE standards, population, setting, outcomes reported, and strategies used to evaluate OSCE outcomes. Where relevant, the authors of included studies will be contacted for clarification or to address missing information. Authors of papers will be contacted up to 2 times to request missing or additional data, where required. Depending on the characteristics of included studies, extracted results may be grouped to support a subgroup analysis. Reviewers involved in the data extraction process will pilot the data extraction tool by reviewing 5 articles, then meet afterward to discuss any conflicts or data extraction challenges. This will allow reviewers to calibrate their understanding of the data being extracted and to add additional data extraction parameters to the tool, as determined by majority consensus. The tool will be modified and revised as necessary during data extraction from each included paper. Any modifications will be detailed in the full scoping review. Any disagreements between the reviewers will be resolved through discussion or consultation with a third reviewer. The final data extraction tool will be made available with the final publication of the scoping review.

### Data Analysis and Presentation

Data analysis will involve mapping and summarizing evidence on how OSCEs are operationalized in health care education programs, in line with the aims and objectives of the scoping review. The data will be presented graphically using figures, charts, and tables to address the primary review question and subquestions. Descriptions of the included studies will be narratively summarized based on the following characteristics of each study: type of record, source, and publication year, study aim, study design, populations, and sample size. The information will be analyzed to identify gaps in study types and geographic context for further research.

Narrative descriptions will describe the characteristics of the participating health care education programs in the studies, educational theoretical perspectives (if reported), and details of OSCE development and implementation. In addition, studies reporting the effectiveness of OSCEs and the competency-based learning outcomes measured will be tabulated. For studies reporting the views and experiences of OSCEs, an analysis of key concepts and themes will also be presented in a tabulated format. This information will be presented for comparative purposes to inform potential future approaches to the application of best practice standards specific to the operationalization of OSCEs and/or further research in the field.

### Ethical Considerations

Ethics approval is not required because the analysis will use published literature and does not involve primary data collection or secondary analysis of data from human participants [22].

## Results

As of July 2026, a search for existing reviews in the PubMed, PROSPERO, Cochrane Database of Systematic Reviews, and JBI Evidence Synthesis databases has been conducted, yielding no current or in-progress reviews on our specific topic. An initial search feasibility assessment has been conducted to ensure sufficient articles for a robust review. The scoping review data collection, extraction, and analysis will be conducted over the 12 months following the literature search. Despite widespread use and adaptation of OSCEs, the authors anticipate identifying significant variances in approaches to OSCEs in health care education. We anticipate completed manuscript submission for publication in the summer of 2027.

## Discussion

### Anticipated Findings

Due to the limited availability of current research within this realm of investigation, the authors hypothesize that existing literature will demonstrate substantial variability and inconsistencies in the operationalization of OSCEs across health care education programs, with limited alignment with established standardized competency-based frameworks. As health care licensure moves toward more generalist competency-based entry-to-practice standards, it is imperative to explore how educational institutions approach developing, implementing, and evaluating OSCE assessments that support readiness for practice.

### Conclusions

OSCEs represent one strategy used in health care education to evaluate student competency and readiness to practice. The authors anticipate that this review will identify variability and inconsistency in the operationalization of OSCEs. By identifying both common and unique elements in OSCE approaches, this review can inform future recommendations for OSCE design, development, and evaluation.

## Abbreviations

ASPE: Association of Standardized Patient Educators
INACSL: International Nursing Association for Clinical Simulation and Learning
NP: nurse practitioner
OSCE: objective structured clinical examination
PRISMA: Preferred Reporting Items for Systematic Reviews and Meta-Analyses
PRISMA-ScR: Preferred Reporting Items for Systematic Reviews and Meta-Analyses extension for Scoping Reviews
vOSCE: virtual objective structured clinical examination
